# Analysis of the Cost-effectiveness of Photodynamic Therapy in Early Stage Lung Cancer

**DOI:** 10.1155/DTE.6.9

**Published:** 1999

**Authors:** Harubumi Kato, Tetsuya Okunaka, Takaaki Tsuchida, Hiroshi Shibuya, Shiro Fujino, Kyoko Ogawa

**Affiliations:** 1 First Department of Surgery Tokyo Medical University 6-7-1, Nishishinjuku, Shinjuku-ku Tokyo 160-0023 Japan; 2 Faculty of Economics Chuo University 742-1, Higashinakano, Hachiouji-shi Tokyo 192-0393 Japan

## Abstract

*Methods* A cost-effectiveness analysis was carried out for photodynamic therapy (PDT) performed in early stage lung cancer cases, which by definition have no lymph node metastasis. The alternative treatment method was lobectomy, which conventionally would have been the first choice of treatment. Costs (*C*) and effectiveness (*E*) both of the PDT group and operation group were compared. Effectiveness was determined using quality adjusted life years saved (QALYs) which is the 5-year survival rate adjusted in terms of the quality of life of the patient, and the cost-effectiveness rate was obtained based on the costs of treatment methods during the patient's stay in the hospital. Health care costs, including drugs, were calculated according to the 1992 National Health Insurance list in yen. Costs which were non-reimbursable by the public insurance system, such as for special rooms and sun block cream, were also expressed in yen.

*Results* The total cost of the operated group was ¥1,793,832 and that for the PDT group was ¥1,017,104. The cost-effectiveness rate of the operated group, that is the average cost of treatment per postoperative living month, was ¥37,537, while that of the entire PDT group was ¥30,003. This indicates that the cost-effectiveness rate for the operated group is apparently 1.3 times higher than that of the PDT group. The monthly cost-effectiveness rate for the PDT group of lesions smaller than 2 cm was ¥25,533. Therefore the cost in the operated group is 1.5 times higher.

*Conclusions* This study demonstrated the merits of PDT for early stage lung cancer from the point of view of cost-effectiveness.


*Diagnostic and Therapeutic Endoscopy*, Vol. 6, pp. 9–16
Reprints available directly from the publisher
Photocopying permitted by license only

© 1999 OPA (Overseas Publishers Association) N.V.
Published by license under
the Harwood Academic Publishers imprint,
part of The Gordon and Breach Publishing Group.
Printed in Malaysia.

### Analysis of the Cost-effectiveness of Photodynamic Therapy in Early Stage Lung Cancer

HARUBUMI KATO[a,*], TETSUYA OKUNAKA[a], TAKAAKI TSUCHIDA[a], HIROSHI SHIBUYA[a], SHIRO FUJINO[b] and KYOKO OGAWA[b]

[a]*First Department of Surgery, Tokyo Medical University, 6-7-1, Nishishinjuku, Shinjuku-ku, Tokyo 160-0023, Japan;* [b]*Faculty of Economics, Chuo University, 742-1, Higashinakano, Hachiouji-shi, Tokyo 192-0393, Japan*

*(Received in final form 13 May 1999)*

***Methods*** **A cost-effectiveness analysis was carried out for photodynamic therapy (PDT) performed in early stage lung cancer cases, which by definition have no lymph node metastasis. The alternative treatment method was lobectomy, which conventionally would have been the first choice of treatment. Costs (*C*) and effectiveness (*E*) both of the PDT group and operation group were compared. Effectiveness was determined using quality adjusted life years saved (QALYs) which is the 5-year survival rate adjusted in terms of the quality of life of the patient, and the cost-effectiveness rate was obtained based on the costs of treatment methods during the patient's stay in the hospital. Health care costs, including drugs, were calculated according to the 1992 National Health Insurance list in yen. Costs which were non-reimbursable by the public insurance system, such as for special rooms and sun block cream, were also expressed in yen.**

***Results*** **The total cost of the operated group was ¥1,793,832 and that for the PDT group was ¥1,017,104. The cost-effectiveness rate of the operated group, that is the average cost of treatment per postoperative living month, was ¥37,537, while that of the entire PDT group was ¥30,003. This indicates that the cost-effectiveness rate for the operated group is apparently 1.3 times higher than that of the PDT group. The monthly cost-effectiveness rate for the PDT group of lesions smaller than 2 cm was ¥25,533. Therefore the cost in the operated group is 1.5 times higher.**

***Conclusions*** **This study demonstrated the merits of PDT for early stage lung cancer from the point of view of cost-effectiveness.**

*Keywords:* Photodynamic therapy, Lung cancer, Cost-effectiveness

*Abbreviations:* QALYs, quality adjusted life years saved; PDT, photodynamic therapy

* Corresponding author. Tel.: 813-3342-6111, ext. 5071. Fax: 813-3349-0326.

### INTRODUCTION

Lung cancer remains the leading cause of cancer death in most Western countries. Despite thousands of clinical trials, the introduction of new approaches to management, and improvements in supportive care, the outlook for most patients diagnosed with carcinoma of the lung remains poor. The rising demand for health care services coupled with static or decreasing resources with which to pay for them has resulted in increasing interest in the economic analysis of medical interventions [1–3]. In this situation, the merits of new cancer therapy are now compared not only in terms of effectiveness but also cost of treatment.

Photodynamic therapy (PDT), a relatively new modality used in the treatment of cancer, has gained considerable acceptance in the past decade [4,5]. A wide variety of malignancies have been treated by this method and according to literature, over 3000 patients worldwide have been treated with PDT [6]. In Japan, PDT with photofrin and excimer dye laser obtained government approval in October 1995 and finally obtained national insurance reimbursement status in April 1996 [7].

This article analyzes the cost-effectiveness in Japan of the new cancer treatment method, PDT, for early stage lung cancer.

### MATERIALS AND METHODS

### Estimate of Costs

The following basic assumptions were made:

(1) Disease: early stage lung cancer (TisN0M0) [8].
(2) Treatment method: curative lobectomy or PDT. In cases of surgery, resection of only one lobe.
(3) Period of hospital stay: 40 days for the operation group (including 3 days in the intensive care unit), 14 days for the PDT group.
(4) No combined chemotherapy or radiotherapy.
(5) Complete curability must be obtained with one surgery procedure or one session of PDT. No recurrence.
(6) Costs of treatment for accompanying diseases are not included in the study.

The standard regimen was established based on the above assumptions, and costs of treatment were obtained by adding the various costs. In this study, the costs estimated include only remuneration costs and other costs paid by the patients during hospital stay and do not include costs after discharge. Because of the typically high age of the subjects, many cases had other diseases such as gastric ulcer and high blood pressure, and treatment for such accompanying diseases was often performed simultaneously. However, such costs were excluded from this study. Total costs can be expressed as follows:

$$C = \mathrm{C1} + \mathrm{C2} + \mathrm{C3} + \mathrm{C4} + \mathrm{C5} + \mathrm{C6} + \mathrm{C7} + \mathrm{C8},$$

where

C1 = cost of medication,
C2 = cost of injections,
C3 = cost of examination,
C4 = cost of diagnostic imaging,
C5 = cost of treatment,
C6 = cost of surgery and anesthesia,
C7 = cost of diagnosis and hospital stay, and
C8 = cost for special (individual) rooms.

Costs C1–C7 are all reimbursable by the public health insurance and C8 must be covered by the patient.

Costs Cl–C8 for the operation group (operated) and the PDT group were estimated as follows. Lobectomy was performed in the operated group and PDT was performed in the PDT group. Since the comparison of costs was made at the point of 5-year survival, in 1993, costs were computed according to the 1992 Ministry of Health and Welfare guidelines [9,10].

*(1) C1 = cost of medication*

The prices of drugs used and cost of compounding are shown in Table I. Based on accumulated data, the average overall cost of drug administration was calculated to be ¥20,000. The PDT group was assumed to receive 30 cc of 4% xylocaine on the day of treatment, 30 cc of 4% xylocaine, 3 tablets of antibiotic preparation per day and 3 tablets of

TABLE I Cost of medication (in yen)

| Operated group | | PDT group | |
|---|---|---|---|
| Basic prescription fee | 420 | Basic prescription fee | 420 |
| Prescription fee | 930 | Prescription fee | 150 |
| Antibiotics, analgesics, antipyretic, anti-inflammatory, anti-bronchoconstrictor, laxative, etc. | 18,650 | Topical anesthesia | 980 |
| | | Antibiotics | 1310 |
| | | Anti-inflammatory | 320 |
| | | Sun block cream | 1000 |
| Total | 20,000 | Total | 4180 |

TABLE II Cost of injections (in yen)

| Operated group | | PDT group | |
|---|---|---|---|
| i.v., i.m. injection | | i.v., i.m. injection | |
| Antibiotics(2 types), diuretics, bronchial mucolytics, blood substitute, analgesics, antiallergics, cholinergic blocker, parasympathomimetics, etc. | 20,000 | Porfimer sodium | 427,000 |
| | | Glucose fluid | 130 |
| | | Atropine sulfate | 100 |
| i.v. drip injection | | i.v. drip injection | 290 |
| Antibiotics(2 types), bronchial mucolytics, blood substitute, $H_2$ receptor antagonist, eupeptic agent, electrolyte transfusion, blood plasma substitute, steroid, extracorporeal circulation diluent, gastric acid inhibitor, diuretics, antihypertensives, antiulcerative, antiallergics, hemostatics, etc. | 120,000 | Electrolyte fluid | |
| i.v. drip route (¥930 × 4) | 3720 | i.v. drip route | 930 |
| Total | 143,720 | Total | 428,450 |

analgesic anti-inflammatory per day for 3 days on which follow-up bronchoscopy was performed, and sun block cream. Since sun block cream is not an NHI listed drug, the price for an over-the-counter sun block cream was used.

*(2) C2 = cost of drug injection (Table II)*

The cost of drug injection consists of costs for the intravenous (i.v.) injection, subcutaneous injection and i.v. drip injection. The cost of i.v. drip injection constitutes a large part of the costs of the operated group. It was assumed that patients postoperatively receive a combination of two types of antibiotic preparations; blood substitute; electrolytic transfusion; liver disease, allergies and antiulcer drugs; 6 times i.v. drip injection or intravenous injection, and the costs for other drugs were added. Calculation for the PDT group was based on their receiving porfimer sodium, and 500 ml electrolyte transfusion to maintain i.v. lines and one ampule of atropine sulfate on the day of treatment. The price of porfimer sodium was ¥427,000, established by The Ministry of Health and Welfare.

*(3) C3 = cost of examination (Table III)*

Patients in the operated group normally stay in the intensive care unit (ICU) for 3 days after the operation. The costs during this period are charged as the ICU fee, and thus are not included in this section. It is not necessary to perform thoracotomy for any examination in the PDT group, but it requires 3 bronchoscopy examinations, including bronchial toilet procedures, the costs of which are included here.

*(4) C4 = cost of diagnostic imaging (Table IV)*

Fewer chest X-rays were required for the PDT group.

*(5) C5 = cost of treatment (Table V)*

Since the cost of bronchial toilet after PDT is included in C3, the cost of treatment for the PDT

TABLE III Cost of examination (in yen)

| | Operated group | PDT group |
|---|---|---|
| Basic examination | 52,000 | 18,200 |
| Basic evaluation | 9000 | 4500 |
| Hematological | | |
| Routine examination | 300 | 300 |
| Peripheral blood data | 350 | 350 |
| Fibrinogen amount | 1850 | 1850 |
| Fibrinolytic products | 1350 | 1350 |
| Biochemical | | |
| Protein demarcation | 350 | 350 |
| Blood gas | 6000 | 8000 |
| Ferritin precision | 2100 | 2100 |
| Aldosterone | 3000 | 3000 |
| Renin | 2150 | 2150 |
| Biochemical evaluation | 1100 | 1100 |
| Immunological | | |
| HCV antibody | 2500 | 2500 |
| Pathological | 46,100 | 20,100 |
| Cardiopulmonary | 6100 | 5000 |
| Monitoring after operation | 11,570 | 0 |
| Clearance test | 4500 | 4500 |
| Endoscopic | | |
| Bronchofiberscopy | 15,000 | 15,000 |
| Follow-up | | 27,000 |
| Drug etc. | 2680 | 8040 |
| Total | 168,000 | 125,390 |

TABLE IV Cost of diagnostic imaging (in yen)

| | Operated group | PDT group |
|---|---|---|
| Roentgenography | | |
| Basic diagnostic fee | 20,000 | 9800 |
| Films | 4380 | 1460 |
| Lung tomography | 5280 | 5280 |
| Films | 4890 | 4890 |
| Bone scintigram | 25,000 | 25,000 |
| Diagnosis | 3750 | 3750 |
| Films, drugs | 35,160 | 35,160 |
| CT scan | 10,450 | 10,450 |
| Diagnosis | 3750 | 3750 |
| Films, drugs | 22,400 | 22,400 |
| Angiogram | 13,050 | 0 |
| Total | 148,110 | 121,940 |

group here is 0. Table V shows the breakdown of the treatment for the operated group. Placement of an indwelling catheter, ultrasonic nebulizer, and oxygen inhalation equipment during the patients' stay in the ICU is included in the ICU cost, thus the cost

TABLE V Cost of treatment (in yen)

| | Operated group | PDT group |
|---|---|---|
| Postoperative treatment of the wound | 4050 | |
| Drainage | 630 | |
| Oxygen inhalation | 1950 | |
| Oxygen | 3970 | |
| Indwelling catheter | 500 | |
| Continuous gastric drainage | 420 | |
| Tube | 750 | |
| Intermittent positive pressure breathing | 1600 | |
| Drug, oxygen | 70 | |
| Nebulizer drug | 360 | |
| Ultrasonic nebulizer drugs | 240 | |
| Local anesthesia during drain placement | 70 | |
| Total | 14,610 | 0 |

TABLE VI Cost of surgery and anesthesia (in yen)

| | Operated group | PDT group |
|---|---|---|
| Cost of procedure | | 87,000 |
| Lobectomy | 319,000 | |
| Automatic suture | 27,000 | |
| Drugs | 65,440 | |
| Cost of anesthesia | | |
| Closed circulatory systemic anesthesia (5 h) | 91,000 | |
| Epidermal anesthesia | 7500 | |
| Continuous injection of anesthesia | 800 | |
| Subcutaneous arterial oxygen monitoring | 1500 | |
| Thoracic epidermal block | 15,000 | |
| Drugs | 1610 | |
| Total | 528,850 | 87,000 |

of treatment for this group is limited to costs for drugs, catheter and oxygen.

*(6) C6 = cost of operation and anesthesia (Table VI)* The cost of operation includes lobectomy, use of one automatic suture device, anesthesia, antibiotics, and local hemostatic agents, etc. Concerning the anesthesia, it was assumed that closed circulatory systemic anesthesia is performed for 5 h, in addition to epidural anesthesia, monitoring of subcutaneous arterial oxygen saturation and neural block.

It was decided to estimate the running cost of the excimer dye laser and the technical charge. Assuming that one excimer dye laser costs ¥40,000,000 and is used for 6 years, the annual depreciation is ¥6,670,000. The running cost for 1 year can be obtained by adding the maintenance fee (routine checkup fee, part exchange fee, janitorial fee, etc.) of ¥2,500,000, laser dye of ¥300,000, and laser gas of ¥200,000 to the above ¥6,670,000. Assuming that 50 patients will undergo this procedure per year, the running cost for one patient is obtained by dividing the running cost for one year by 50 plus ¥40,000 for the light transmission fiber and ¥1000 for electricity (¥234,400). Adding these up, the total cost of the procedure is assumed to be ¥500,000. In addition, since PDT only requires local anesthesia for bronchofiberscopy, which is already included in the examination category cost, the cost of local anesthesia is not included in this section.

*(7) C7 = cost of diagnosis and hospital stay (Table VII)*

The cost of diagnosis for both operated and PDT groups includes ¥1950 for initial examination and ¥5000 for management of the records of malignant tumor treatment.

Concerning the costs related to hospital stay, it was assumed that patients receive regular food, use a regular bed and pajamas and receive high quality nursing care. Including the preoperative period, the length of stay in the hospital was assumed to be 40 days for the operated group and 14 days for the PDT group. However, the operated group has an extra charge for stay in the ICU for 3 days, for which time the cost of hospital stay (apart from food costs) was excluded.

*(8) C8 = cost for special rooms*

Special room costs differ greatly depending on individual hospitals, and the number of patients per room. This study applied the average extra cost for special rooms of ¥4356 reported in the 1991 investigation of room cost by the Insurance Department of the Ministry of Health and Welfare. This cost cannot be obtained for stays in the ICU. C8 for the operated group was ¥161,172 and that for the PDT group was ¥60,984.

### RESULTS

Table VIII shows the total cost for the operated group and the PDT group from C1 to C8. The total cost of the operated group was ¥1,793,832 and that for the PDT group was ¥1,017,104.

In this analysis the end point of the treatment is set as 5-year survival and the cost-effectiveness is estimated on a monthly basis. This study used

TABLE VII Cost of diagnosis and hospital stay (in yen)

| | Operated group | PDT group |
|---|---|---|
| Initial examination fee | 2300 | 2300 |
| Management of malignant tumor treatment | 5000 | 5000 |
| Medical management fee at the time of admission | | |
| Days 1–14 | 62,810 | 79,940 |
| Addition for ICU | 186,000 | |
| Days 15 up to 1 month | 59,200 | |
| 1–2 months | 25,800 | |
| Basic nursing fee | 124,320 | 47,040 |
| S-2 class nursing fee, days 1–30 | 56,430 | 29,260 |
| days 31– | 19,800 | |
| Room charge | 67,710 | 25,620 |
| Total | 609,370 | 189,160 |

TABLE VIII Total cost of operated group and PDT group (in yen)

| | Operated group | PDT group |
|---|---|---|
| C1 = cost of medication | 20,000 | 4180 |
| C2 = cost of injections | 143,720 | 428,450 |
| C3 = cost of examination | 168,000 | 125,390 |
| C4 = cost of diagnostic imaging | 148,110 | 121,940 |
| C5 = cost of treatment | 14,610 | 0 |
| C6 = cost of surgery and anesthesia | 528,850 | 87,000 |
| C7 = cost of diagnosis and hospital stay | 609,370 | 189,160 |
| C8 = cost for special rooms | 161,172 | 60,984 |
| Total | 1,793,832 | 1,017,104 |

retrospective data from Tokyo Medical College. The 5-year survival for the operated group (39 cases) was 91% and that of the PDT (total 36 cases) group was 95.1%. PDT cases with lesions under 2 cm in maximum dimension (30 cases) obtained a 5-year survival rate of 100% [7,11]. Causes of death for the operated group include postoperative complications such as pneumonia, pyothorax, and infection. The average survival was 54.6 months for the operated group, 51.06 months for the entire PDT group.

The quality of life of patients in the operated group as measured by percent vital capacity (%VC) decreased by 10%. Since no lobectomy was performed in the PDT group, the %VC remained at 100%. The quality of life was estimated in terms of the quality of adjusted life years (QALYs). In the post-procedural period of observation of 5 years QALYs of patients were 49.14 months for the operated group, 51.06 months for the entire PDT group and 60 months for PDT cases with lesions less than 2 cm in maximum dimension. QALY units were used to indicate effectiveness in this study.

Using the costs and effectiveness discussed above, the rates of cost-effectiveness of the two groups were compared. The cost-effectiveness rate of the operated group, that is the average cost of treatment per postoperative living month, was ¥37,537, while that of the entire PDT group was ¥30,003. This indicates that the cost-effectiveness rate for the operated group is apparently 1.3 times lower than that of the PDT group. The monthly cost-effectiveness rate for the PDT group of lesions smaller than 2 cm was ¥25,533. Therefore the cost in the operated group is 1.5 times higher.

### DISCUSSION

Increasing numbers of early stage lung cancer cases are being detected as a result of improved survey and diagnostic techniques. Despite the possibility of curative resection in such cases, many patients are frequently at high surgical risk because of coexisting chronic obstructive pulmonary disease or cardiovascular disease. Considering the quality of life of the patients, it is essential to preserve lung tissue by treating the initial early stage lung cancer as conservatively as possible. The introduction of PDT has provided a new therapeutic alternative to surgery.

PDT aims to treat cancer through the photochemical reaction of the drug which is specifically taken up by cancer cells by (1) intravenous injection and (2) shining low-power laser photoradiation of the lesion. The authors began investigation of these techniques in collaboration with Dr. Thomas Dougherty in 1978 and demonstrated their effectiveness in both diagnosis and treatment in canine lung cancer models, and subsequently applied these methods in clinical cases in 1980 [12,13]. Since then, 370 cases, including 240 lung cancer patients (283 lesions), were treated with PDT in our institution. There were several cases of 5-year survival, including the first such case in the world treated by PDT alone [14].

PDT has no serious side effects apart from sensitivity to sunlight. It was also apparent that it not only reduces the psychological and physical burden on patients but also is a therapy with high economical cost-effectiveness in the Japanese medical environment. In addition, PDT has the advantage of not excluding other therapeutic possibilities, and even cases in which a satisfactory result was not obtained with PDT can still subsequently undergo surgery. PDT obtained a 100% 5-year survival rate for early stage superficial squamous cell carcinomas, with a maximum dimension of less than 2 cm, indicating the possibility of taking the place of surgery as the first treatment of choice for cancer at this stage. At present the rate of detection of lesions at such an early stage is very low, but it is expected that the frequency of detection will increase with the spread of sputum cytology survey, thus improving the results of lung cancer treatment.

This study attempted to evaluate and compare PDT, a new lung cancer treatment method, with conventional surgical operation in terms of QALYs.

The data reflect the pattern of common practice in Japan. Both the PDT example and the example of surgical treatment represent absolutely standard approaches in Japan. This means that the periods of hospitalization for both procedures, as well as the numbers and amounts of concomitantly used drugs for the latter, are greater than in North America or Europe. Effectiveness can be evaluated using the 5-year survival rates for both therapeutic methods, but the decrease of pulmonary function, especially vital capacity after lobectomy, was also taken into account. Resection of a part of the lung affects the quality of postoperative life.

Lobectomy used to be performed for treatment of tuberculosis, which resulted in longer survival time for patients. However, cases which underwent lobectomy showed subsequent decrease in pulmonary function, requiring home oxygen therapy in some cases. In addition, as a result of lowered respiratory function, the risk of respiratory insufficiency due to infection becomes high. In the treatment of cancer, lobectomy can cause similar complications as in tuberculosis cases. Therefore, it was decided to use the condition of the post-operative pulmonary function (%VC) as an indication of quality of prognosis. If lobectomy is not performed, the pulmonary function can be maintained at the preoperative level postoperatively (%VC = 100%), but lobectomy in some extreme cases can result in death. It was attempted to evaluate the quality of life based on %VC.

Average lengths of survival were adjusted using the rate of decrease in %VC as an index of quality of life, and this was used to indicate true effectiveness, expressed in terms of QALYs. This is in principle similar to Karnofsky's performance status, in which method QOL is indicated by the patient's ability to perform various activities [15]. In these analyses the number of data is often limited and analysis is restricted to some extent. Analysis of a long-term model could be possible if data on multiple cancer, rate of recurrence, rate of survival, etc. were available.

Even in Japan, where its people have lived under the protection and security of its health plan, the country's health budget is now in jeopardy. For instance, in the treatment for cancer, there was a great tendency to turn to surgery alone. However, the current way of treatment emphasizes the patient's needs and QOL greatly while maintaining cost-effectiveness. As a result of this "new" tendency, the number of minor invasive treatment cases, such as PDT, bave been increasing. With the development of second generation photosensitizers, which produce no skin photosensitivity, patients treated with PDT may now possibly be admitted for a shortened term; thereby contributing to the cost effectiveness of Japan's health budget.

*Acknowledgment*

The authors would like to thank Professor J.P. Barron of Tokyo Medical College for his review of the manuscript.

### *References*

[1] Task Force on Principles of Economic Analysis of Health Care Technology. Economic analysis of health care technology: A report on principles. *Ann. Intern. Med.* 1995; **122**: 61–70.
[2] Smith, T.J., Hillner, B.E., Neoghbour, D.M., McSorley, P.A. and Chevalier, T.L. Economic evaluation of a randomized clinical trial comparing vinorelbine, vinorelbine plus cisplatin, and vindesine plus cisplatin for non-small-cell lung cancer. *J. Clin. Oncol.* 1995; **13**: 2166–2173.
[3] Micheal Boyer. The economics of lung cancer. *Lung Cancer* 1996; **14**: 13–17.
[4] Kato, H. and Okunaka, T. Photodynamic therapy in early tumors. In: Hetzel M. (Ed.) *Minimally Invasive Techniques in Thoracic Medicine and Surgery*. London: Chapman and Hall Medical, 1995: pp. 149–172.
[5] Furuse, K., Fukuoka, M. and Kato, H. A prospective phase II study on photodynamic therapy with Photofrin II for centrally located early stage lung cancer. *J. Clin. Oncol.* 1993; **11**: 1852–1857.
[6] Marcus, S.L. and Dugan, M. Global status of clinical photodynamic therapy: The registration process for a new therapy. *Lasers Surg. Med.* 1992; **12**: 318–324.
[7] Kato, H., Okunaka, T. and Shimatani, H. Photodynamic therapy for early stage bronchogenic carcinoma. *J. Clin. Laser Med. Surg.* 1996; **14**: 235–238.
[8] General Rules for Clinical and Pathlogical Record of Lung Cancer: The 4th edition, The Japanese Lung Cancer Society, November, 1995.
[9] NHI price list: Notification No. 30 of the Ministry of Health and Welfare, March 12, 1992.
[10] Medical fee charge list: Notification No. 40 of the Ministry of Health and Welfare, March 19, 1992.

[11] Konaka, C., Okunaka, T. and Kato, H. Combined use of photodynamic therapy and surgery. *Ann. Thorac. Cardiovasc. Surg.* 1995; **1**: 55–59.

[12] Dougherty, T.J., Laurence, G. and Kaufman, J.H. A. Photoradiation in the treatment of recurrent breast carcinoma. *J. Natl. Cancer Inst.* 1979; **62**: 231–237.

[13] Hayata, Y., Kato, H. and Konaka, C. Hematoporphyrin derivative and laser photoradiation in the treatment of lung cancer. *Chest* 1982; **81**: 269–277.

[14] Kato, H., Konaka, C. and Ono, J. Five-year disease-free survival of a lung cancer patient treated only by photodynamic therapy. *Chest* 1986; **90**: 768–770.

[15] Karnofsky, D.A. The clinical evaluation of chemotherapeutic agents in cancer. In: MacLeod, C.M. (Ed.) *Evaluation of Chemotherapeutic Agents*. New York: Columbia Univ. Press, 1949: pp. 191–205.

